# Queer Facts about Teeth

**Published:** 1894-07

**Authors:** 


ARTICLE VI.

### QUEER FACTS ABOUT TEETH.

ESPECIALLY MANKIND'S AND INCIDENTALLY THOSE OF BIRDS AND FISHES.

"You will often hear it said that man is by nature a carnivorous animal, as is shown by the incisors or so-called 'canine' teeth with which he is provided, but it is not true. Human beings are carnivorous only by habit, and not by nature." It was Osteologist Lucas, of the Smithsonian, who said so, and he added:

"You see, it is always from an animal's teeth that the diet intended for it by nature is judged. But the fact that it has incisors does not prove that it is carnivorous. There are plenty of purely vegetable-eating beasts which have well developed incisors. Take the monkey, for example. Monkeys' incisors are much more develoved than those of man, but they are exclusively fruit eaters, the incisors being useful merely for fighting. We, who are descended from anthropoid apes, still have the canine teeth, but for the reason that we no longer employ them for combative purposes, they have become smaller. Man was originally frugivorous, presumably, though the time when he first began to eat meat must have been very far back, judging from the remains of extinct mammals found in the caves among the ashes of his cooking fires, which burned hundreds of thousands of years ago.

"It is by the teeth of mammals that they are most readily classified, inasmuch as the dentition illustrates the food of the animal and the general habits which necessarily depend upon its manner of procuring food. The teeth by which these things are determined are not the incisors, but the molars or grinders. A mammal usually has several kind of teeth in its jaws. Take the monkey, for example. Its front teeth are for catching up and nipping little things. With them it catches and kills its parasites, the fleas, as does likewise the dog. Its incisors are for fighting, although in carnivora they are employed to pierce the flesh deeply so as to open the veins and bleed the victim to death."

"Thus, you will find that a tiger will know by instinct where to strike the jugular vein of an ox, of the location of which, I dare say, that you yourself are not very definitely aware. Behind the incisors in the monkey, as well as in man, are found the pre-molars, which are for cutting up what is to be swallowed, while the molars themselves perform the grinding process. It is really wonderful, you will observe, how admirably the entire apparatus is adapted for the purpose in view. You find the most remarkable development of the incisors or canines in the extinct saber-toothed tiger, contemporary with the cave men, which had two knife-shaped teeth in its upper jaw so huge that it often died in consequence of catching them in the lower jaw, being thus unable to close its mouth. Each of these weapons had a keen saw on its inner edge.

The tusks of an elephant are the upper incisors of the beast. They are not intended for chewing, however, but for defense. You find all through creation the most astonishing adaptation of the teeth to necessity. You are familiar, of course, with the mighty ivory lance of the narwhal, ten or twelve feet in length, and strong and sharp enough to be driven through the side of a ship. The lance is simply the left upper incisor of the mammal. Once in a while, by a freak, both of the upper incisors will be developed in the narwhal, so that it is equipped with two spears, instead of one. The tooth in this case is designed for a weapon in fighting. The female has no lance.

"Look at the sawfish. The entire length of its saw, which is a prolongation of the nasal process, is fringed with teeth. Again you have a weapon merely, the manner of the creature being to strike right and left for the purpose of wounding its prey. In mammals, however, the teeth are restricted to the jaw-bones. Lizzards and snakes have them on the bones of the palate as well. True, bony teeth are peculiar to animals which have backbones. The most elaborate dental apparatus known belongs to the sea urchin, whose jaws are composed of forty pieces, moved by forty separate muscles. Snails have a sort of ribbon with which they rasp their food as with a file. Anteaters, though they are mammals, have no teeth at all; but they get there just the same, having no need to chew their prey. The whalebone whale is a mammal that has no teeth, its practice being to swallow its food whole."

"The biggest of fresh water fishes—the 'arapaima' of the Amazon, in South America, which grows to six feet in length—has teeth on its tongue, so that the latter resembles the file, and is used as such. Some kinds of trout also have the same peculiarity. Fishes that swallow their prey entire have their teeth so supported on flexible bases as to bend backward, but not forward, in order that their victims shall not escape after they have been seized."

"In ages gone by there were ferocious sharks, such as would make a mouthful of you without blinking, seventy feet in length. Plenty of their teeth have been found which are five inches long, whereas the biggest of the teeth belonging to sharks that exist at the present day are one and a-half inches long. Speaking of extinct creatures reminds me to say that all of the early birds—those of early geologic times, that is—had teeth, with which they captured the early worms of the same period. Being descended from reptiles, it is natural that they should possess a dental equipment, but when they ceased to be carnivorous they had no teeth any longer."

"Certain animals have teeth which grow during all their lives. The rat and the squirrel are examples of this. Our own teeth are developed from pulps, which are absorbed and disappear after the teeth are grown, but in a rat's tooth the pulp is perpetual, and is continually secreting material by which the incisor gains length. Therefore, the animal is obliged to gnaw all the time to keep them ground down to the proper length. It is commonly imagined that the rats keep gnawing from pure cussedness, but such is not the case."

"Sometimes it happens that the beast's upper and lower incisors do not meet properly, so that it is unable to gnaw, and its teeth keep growing around in a spiral. Cases have been known where a rat's tooth grew in this manner through its skull so as to pierce the brain and kill the unfortunate. The biggest teeth I know of are those of the mastodon, which we have in the shape of fossils. One advantage about teeth is that they are harder than almost anything else in nature, and will last longer, so that they may be picked up in an excellent state of preservation ages after the animals to which they originally belonged are dead."

"You often hear of rendering a rattlesnake harmless by pulling out its fangs. Then, again, you read of cases where a serpent so treated has bitten persons fatally. The reason for this is that a poisonous snake is deprived only temporarily of its venomous powers by the extraction of the two incisors in the upper jaw, at the bases of which are the poison glands. Of course, you know that the fangs are hollow, so that when the animal strikes the venom gushes through them into the flesh of the person struck. Now, by drawing the two teeth the snake may be rendered harmless for a few weeks, but after a short time the two teeth just behind the original fangs move up and take their places, making connections with the poison glands, and thus becoming poison fangs as good and effective as the old ones."—*Washington Star*.

